# Pyorrhœa Alveolaris

**Published:** 1892-03

**Authors:** Edgar F. Stevens

**Affiliations:** Boston, Mass.


### PYORRHŒA ALVEOLARIS.[1]

BY DR. EDGAR F. STEVENS, BOSTON, MASS.

WRITERS of twenty years ago seldom mention pyorrhœa alveolaris as occurring in patients under thirty, while at the present time we see indications of it in those under eighteen. Why this is the case is not clear to my mind. We find it in patients who live on plain food as often as in those of luxurious diet; in those who are in a good physical condition as frequently as in those of feeble health; in patients suffering from nasal catarrh no more than in those who are not.

Usually, I believe, the trouble has originated from a simple deposition of salivary calculus, which, we know, is first deposited at the margin of the gum, in close proximity to the ducts of the salivary glands. As this deposit is added to, the gum is forced away from the tooth, and the constant irritation caused by this deposit and by forcing food against the gum in mastication soon develops inflammation, nutrition is interfered with, and we have a breaking down of the connective-tissue corpuscles with formation of pus; the teeth become loose, elongated, and forced out of place, especially the tissues about the lower incisors, which become so swollen as to have the appearance of abscess; finally, a breaking down of the bony tissue of the alveolus takes place. This condition often spreads to adjacent teeth, which were, perhaps, free from deposit, and the entire set may be involved. In extreme cases we may have more or less systemic disturbance, as fever, loss of appetite, and digestive trouble, probably largely due to the amount of pus which must be taken into the stomach with food.

In treating these conditions, thoroughness in cleansing is of the greatest importance; in the first place, the deposit must be entirely removed by properly-shaped instruments. It is not well to attempt

---

[1] Read before Harvard Odontological Society, October 29, 1891, by Dr. W. E. Boardman.

to complete the treatment at the first sitting, but be thorough in what is done. After the deposit is dislodged from the root, wash out the pocket with warm water, and if the alveolus is involved, use engine-burs to cut away until healthy bone is found, then rinse with hydrogen peroxide, and after being sure all *débris* is removed, protect the soft tissues with napkins, and with a Donaldson broach wound with cotton apply caustic paste to the socket as far down as can be reached. Wait two or three minutes, then rinse with warm water, and pack tannin and glycerin paste in the socket. If the pocket is very deep, lance from top to bottom; this will not give pain, as the caustic paste has obtunded the parts. If the teeth are very loose, tie with silk ligature, weaving in and out, so as to make a strong support.

In dismissing patient until next appointment (which should be in two or three days), be sure to impress upon his mind the importance of absolute cleanliness on his part; let him rinse his mouth several times a day with a wash containing half a drachm each of carbolic acid and liquid potassæ in a pint of distilled water. For a tonic, something like Wyeth triple phosphates, or cinchonidia, two-grain pills three times a day, and keep the bowels free by cathartics. At the second sitting, carefully remove whatever deposit was not dislodged at first, and inject hydrogen peroxide into the socket, after which burn out the dead or diseased tissue with the caustic paste, rinse with warm water, and pack as before with tannin and glycerin. As a rule, the brush has been used too sparingly, and we can now do a little teaching as to what we mean by thorough and faithful cleaning; this will bear its fruit at the next appointment.

The number of times a patient requires to be seen depends entirely on conditions, but, as a rule, after the gums have been satisfactorily treated and brought into a condition of comfort, it will not be necessary to see the patient oftener than once in three or four months. I do not claim to always cure this trouble, but where the socket has not been extensively destroyed, we can make the parts comparatively healthy.

There is another condition met with where the gum has receded without a deposit being found, which affects particularly the lower incisors and upper and lower cuspids. It is characterized by a retraction of the gum margin while still holding tightly to the root; there are no signs of inflammation, being simply an atrophy of the parts which probably has a constitutional or hereditary origin. Mechanical means to overcome this trouble cannot be used; we must recommend proper cleansing and systemic tonics.

In cases where the posterior teeth have been lost and the anterior teeth have been forced out of line, I would, as a rule, extract.

Sometimes we meet cases where the incisors have elongated and been pushed outward, perhaps the other teeth are in a condition to to be treated and saved, but there is no help for the incisors, for if treatment is commenced they will continue to elongate and spread, and may as well be removed at once.

